# Tracking a Major Egg Allergen to Assess Commercial Food Label Compliance: Towards a Simple and Fast Immunosensing Device

**DOI:** 10.3390/bios12121109

**Published:** 2022-12-01

**Authors:** Maria Freitas, Mariana del Rio, Henri P. A. Nouws, Cristina Delerue-Matos

**Affiliations:** REQUIMTE/LAQV, Instituto Superior de Engenharia do Porto, Instituto Politécnico do Porto, Dr. António Bernardino de Almeida 431, 4200-072 Porto, Portugal

**Keywords:** egg white, Gal d 3, European legislation, electrochemical biosensor, immunosensor, amperometry, food allergy, commercial food, product label

## Abstract

An amperometric immunosensor was developed for the analysis of the major egg-white allergen ovotransferrin (Gal d 3) in commercial food products because the (accidental) intake, skin contact with, and/or inhalation of eggs can lead to severe disorders in allergic individuals. Employing a sandwich-type immunosensing strategy, screen-printed carbon electrodes (SPCE) were biomodified with anti-Gal d 3 (capture) antibodies, and the allergen’s detection was achieved with anti-Gal d 3 antibodies labelled with horseradish peroxidase (HRP). The 3,3′,5,5′-tetramethylbenzidine (TMB)/H_2_O_2_ reaction with HRP was used to obtain the electrochemical (amperometric) signal. An attractive assay time of 30 min and a remarkable analytical performance was achieved. The quantification range was established between 55 and 1000 ng·mL^−1^, with a limit of detection of 16 ng·mL^−1^. The developed method demonstrated good precision (V_x0_ = 5.5%) and provided precise results (CV < 6%). The sensor also detected extremely low amounts (down to 0.010%) of egg. The analysis of seven raw and/or cooked egg and egg-white samples indicated that food processing influences the amount of allergen. Furthermore, to assure the compliance of product labelling with EU legislation, 25 commercial food ingredients/products were analysed. The accuracy of the results was confirmed through an ELISA assay. The stability of the ready-to-use sensing surface for 20 days allows a reduction of the reagents’ volumes and cost.

## 1. Introduction

The chicken/hen’s egg (Gallus gallus domesticus) is a functional food that is widely included in the human diet, constituting a prevalent option as a source of protein and nutritious content (mostly by the edible parts, the egg white and yolk) and providing culinary variety at a low economic cost [1]. Additionally, the choline content has positive effects on memory and the development of the central nervous system in the foetus [2]. Nevertheless, eggs are a major allergy-causing food for children. The edible egg fractions (white and yolk) can cause allergic sensitization; however, allergic symptoms/reactions are mainly triggered by egg-white proteins (ovomucoid, ovalbumin (conalbumin), ovotransferrin, and lysozyme). Thus, the presence of egg in foodstuff and products thereof must be highlighted by assertive labelling, as required by EU legislation (1169/2011) [3]. There is, therefore, a pressing need for the efficient detection of egg allergens. Eggs from other birds (e.g., duck, goose, quail), despite having lower inclusion/consumption, can also be included in daily meals. Thus, in case of hypersensitivity, tracking the presence of egg in food products is a major concern since the only option for consumers remains to eliminate the different types of eggs from their diet [4].

Efficient detection of egg content is conventionally performed using the protein fraction of the sample (e.g., ELISA, immunoassays). In fact, there are still no effective molecular/genetic tests implemented for the analysis of egg allergens (e.g., PCR) due to the low amount of DNA that can be successfully extracted and analysed. Additionally, differences in the DNA of chicken meat and/or chicken eggs content are not significant, and their analysis may lead to questionable results [5,6].

Unfortunately, the analysis of food allergens using robust and reliable methods, with a fast response time, is still a current problem. Modern (bio)sensors are small portable handheld devices that can overcome contemporary food/diet challenges. Additionally, the development of ready-to-use biosensors fits the technological innovation regarding consumer demand [7]. To overcome the drawbacks of the conventional methods (e.g., analysis in centralized laboratories with expensive equipment and expert personnel), the employment of immunosensors with the intended robustness can respond to the need for the onsite tracking of egg allergens in pre-packed food products [8,9,10,11].

Thus far, no electrochemical immunosensors have been reported for the analysis of ovotransferrin (Gal d 3). In the few recently reported immunosensors for the analysis of chicken/hen’s egg allergens, ovomucoid (Gal d 1) was analysed in unprocessed and baked samples using a magnetic immunoplatform [12]. Besides Gal d 1, methods for the analyses of ovalbumin (Gal d 2) have also been reported using a label-free format [13], non-competitive (sandwich) assays [14,15,16], and multiplexed analysis using a microfluidic device [17].

Although current legislation only requires mentioning the presence of egg on the product’s label, a major drawback for hypersensitive persons lies on the presence of egg white and/or yolk [3]. Interestingly, new approaches in the food industry present their product labels referring to the presence of eggs in different formats (liquid, powder, cooked) since food processing can lead to variations in the amount of allergen. Thus, it is possible to present detailed information to the consumer; however, to ensure this information, validation must be carried out. Home-use monitoring devices with fast analysis results can be an advantage to consumers.

The analysis of Gal d 3 in food products is critical since it causes adverse reactions in hypersensitive individuals (even at trace amounts) [18]. Therefore, this allergen was the target analyte of this study, which consisted of the development of an amperometric sandwich-type immunosensor for food safety and quality control of commercial products. The electrochemical biosensor was employed in the analysis of Gal d 3 in a set of 25 commercial food samples (including products with labels that indicated “does not contain egg”, “may contain traces of egg”, “contains egg”, and/or “contains egg white”). Furthermore, the influence of food processing on the allergen amount was verified through the analysis of several raw and/or cooked egg and egg-white samples.

## 2. Materials and Methods

### 2.1. Apparatus and Equipment

A potentiostat/galvanostat (PGSTAT101) and NOVA software (v.1.10) (Metrohm Autolab, Utrecht, The Netherlands) were used to record the chronoamperograms. Screen-printed carbon electrodes (SPCEs, DRP-110, ceramic substrate (L 33 × W 10 × H 0.5 mm), Metrohm DropSens (Oviedo, Spain)) were used as transducers. These electrodes are composed of a three-electrode electrochemical cell: a carbon working electrode (WE, d = 4 mm), a carbon auxiliary electrode (AE), a silver pseudo-reference electrode (RE), and silver connections. A centrifuge (Megafuge 16R Thermo-Heraeus, Thermo Fisher Scientific, Osterode am Harz, Germany) and an electric grinder (Moulinex, France) were employed for sample preparation. The results of the food sample analyses were validated by ELISA, carried out using a multi-mode microplate reader (Synergy HT W/TRF, BioTek Instruments, Winooski, VT, USA) and Gen5 data analysis software (v. 2.0, BioTek Instruments).

### 2.2. Reagents and Solutions

Chicken ovotransferrin (Allergen, Gal d 3, purified from chicken egg white) and a matched antibody pair (unconjugated rabbit anti-Gal d 3 polyclonal antibody (CAb) and peroxidase-conjugated rabbit anti-Gal d 3 polyclonal antibody (DAb-HRP)) were supplied by MyBioSource. Bovine serum albumin (BSA), 3,3′,5,5′-Tetramethylbenzidine (TMB) liquid substrate (TMB-H_2_O_2_ K-Blue reagent), potassium dihydrogenphosphate, potassium hydrogenphosphate trihydrate, and sodium chloride were acquired from Sigma-Aldrich.

Phosphate-buffered saline (PBS), a 0.01 M phosphate solution containing 137 mM NaCl, pH 7.4, was used for both the biosensor construction (B1) and the allergen extraction (extraction buffer) from food samples. The daily working solutions of the biomolecules (standard allergen or food sample and DAb-HRP) were prepared in B1-BSA 0.5% (m/V).

### 2.3. Food Sample Preparation and Allergen Extraction

To evaluate the usefulness of the developed sensor, Gal d 3 was determined in 6 distinct egg-white samples (liquid, powdered, raw, and/or cooked) and a whole egg (white and yolk, raw). Additionally, 25 food products and ingredients with, without, or containing traces of egg were purchased in local supermarkets (Porto, Portugal) to evaluate the sensor’s capability of detecting (trace) amounts of the allergen. The commercial samples were homogenized (grinding), blended (1 g, 10 mL of extraction buffer, vortexed for 5 s), thermally processed (60 °C, 30 min), and centrifuged (5000 rpm for 5 min for the whole sample; additional 5 min at 10,000 rpm for the collected supernatant). The obtained extract was maintained for 1 week at 4–8 °C for the allergen analysis and then kept at −20 °C for long-term storage [10]. The accuracy of the sensor’s results was assessed by comparison with an ELISA assay, with a dilution of 250× (in B1-BSA).

### 2.4. Immunosensor Construction and Detection Strategy

**Figure 1:**
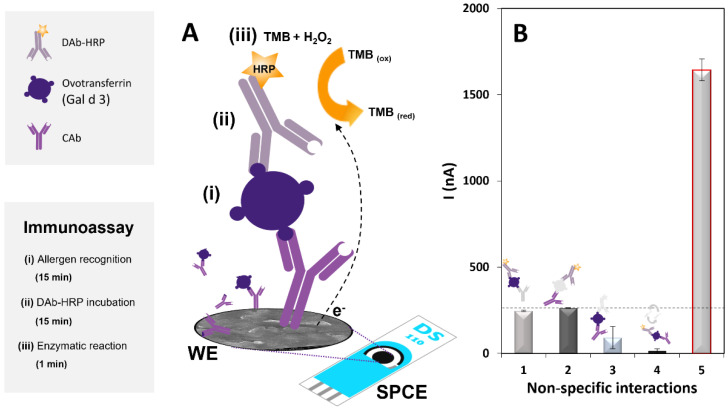
Schematic representation of the electrochemical immunosensor construction. (**A**) SPCE biomodification, immunosensing strategy, enzymatic reaction, and analysis and (**B**) current intensities (I, nA) obtained in the evaluation of the sensor’s performance and non-specific interactions: absence of (1) CAb, (2) allergen, (3) DAb-HRP, (4) TMB-H_2_O_2_, and presence of all the reagents (5).

The main steps of the sandwich-type immunosensor’s construction and the electrochemical detection strategy are schematically represented in Figure 1A.

The optimized protocol consists of the transducer’s biomodification (physical adsorption) by drop-casting 10 µL of a CAb solution (2.5 µg·mL^−1^) onto the SPCE’s WE, which was left in a moist chamber overnight. After washing with B1, the immunoassay was performed as follows:

(i) The antigen (40 µL of standard allergen or food sample solution, prepared in B1-BSA 0.5%) was incubated for 15 min, and once the electrode was washed with B1, (ii) the DAb-HRP solution was added (40 µL, 200× dilution in B1-BSA 0.5%) and incubated for 15 min. The SPCE was then washed with B1 to remove unbound biomolecules, and (iii) a 40-µL aliquot of the TMB-H_2_O_2_ K-Blue reagent solution was added. After 1 min, chronoamperometry (fixed potential of 0 V, applied for 1 min) was used to record the analytical signal [19]. The obtained current (I, the average of the last 10 s) is directly proportional (in the linear range) to the amount of allergen present in the solution. The total assay time was about 30 min, with a hands-on time of 10 min.

All the results are presented as the average and the standard deviation of three replicates.

## 3. Results and Discussion

In an initial study, the suitability of the antibodies, enzyme, and enzymatic substrate was studied, and non-specific interactions were evaluated to confirm the effectiveness of the purposed strategy [20,21]. According to previous works, chronoamperometric detection using HRP and TMB/H_2_O_2_ can be performed after a 1 min enzymatic reaction, where the oxidized form of TMB is electrochemically reduced by applying a potential of 0 V [19,22,23,24]. Thus, the obtained amperograms for the biomolecule interactions (Figure 1B) confirm the adequate performance of the immunosensor in the absence of (1) CAb, (2) allergen, (3) DAb-HRP, and (4) TMB-H_2_O_2_ and in (5) the presence of all the reagents. The obtained results demonstrate that a correct strategy was used since no significant non-specific interaction was observed.

### 3.1. Optimization of the Immunoassay

**Figure 2:**
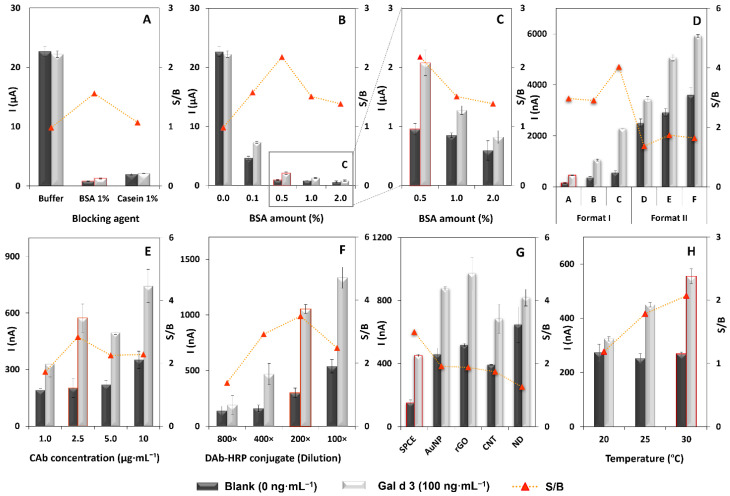
Optimization of the experimental parameters/variables. (**A**) Blocking agent (no blocker (buffer), BSA, and casein); (**B**) BSA amount (0.0, 0.1, 0.5, 1.0, and 2.0% (m/V)); (**C**) amplification of the best results obtained with BSA (0.5, 1.0, and 2.0% (m/V)); (**D**) assay strategy (Format I—step-by-step; Format II—previous mixing of Gal d 3 and DAb-HRP); (**E**) CAb concentration (1.0, 2.5, 5.0, and 10 µg·mL^−1^); (**F**) DAb-HRP dilution (800×, 400×, 200×, and 100×); (**G**) SPCE modification with nanomaterials (bare SPCE vs. SPCE with AuNP, rGO, CNT, and ND); (**H**) temperature (20, 25, and 30 °C). Other experimental parameters: Gal d 3: 0 ng·mL^−1^ (dark grey bars) and 100 ng·mL^−1^ (light grey bars); error bars correspond to the standard deviation of three replicates.

To evaluate the main effects of the biomolecule’s interactions and the required conditions for the sensor’s construction, the influence of distinct parameters/variables was studied to prepare a robust immunosensor for the difficult task of tracking trace amounts of ovotransferrin in foodstuff. The used parameter to choose the most adequate conditions was the signal-to-blank (S/B) ratio, which corresponds to the ratio of the currents obtained for 100 ng·mL^−1^ Gal d 3 (S) and blank (B, 0 ng·mL^−1^ of Gal d 3) solutions. The results are shown in Figure 2. A crucial aspect for the correct performance of the assay is the selection of the solution for the preparation of the standard allergen or food sample and DAb-HRP solutions. Thus, B1, B1-BSA, and B1-casein solutions were tested (Figure 1A), showing that in the absence of a blocking agent (B1), no signal differentiation between the presence/absence of Gal d 3 (S/B ≈ 1) was observed. A similar S/B value was observed when casein was added, but much lower currents were obtained. This is due to excessive blockage of the WE’s surface that hinders the electron transfer. On the other hand, when B1-BSA was used, an S/B ratio above 1 was obtained. The effect of distinct amounts of BSA (0.0, 0.1, 0.5, 1.0, and 2.0% (*m*/*v*)) was then studied. As can be seen in Figure 2B,C, the addition of 0.5% to B1 led to the highest S/B ratio, so this solution was chosen for the subsequent studies. The previous studies were performed using a step-by-step format which corresponds to a 2 h assay (antigen, 60 min + DAb-HRP, 60 min). To minimize the assay time, two assay formats were tested (Figure 2D): Format I (step-by-step), performed in two steps: incubation of (i) antigen for 15, 30, or 60 min and (ii) DAb-HRP for 15, 30, or 60 min, resulting in total assay times of 30, 60, and 120 min (Format I: A, B, C), and Format II, corresponding to a single-step assay that comprises pre-incubation (10 min before use) of antigen and DAb-HRP and subsequent incubation for 30, 60, and 120 min (Format II: D, E, F). Although the Format II assays revealed higher currents, even for lower incubation times, the best S/B ratios were achieved for the Format I assays. The highest S/B ratio was obtained for Format IC, but a significantly longer time (120 min) was needed to obtain the result compared to the other assays (30 and 60 min). The S/B ratios for Formats IA and IB were similar, so Format IA (30 min) was selected to proceed with the work because of the shorter assay time.

After the assay format was chosen, the CAb concentration (1.0, 2.5, 5.0, and 10 µg·mL^−1^) and the DAb-HRP conjugate dilution (800×, 400×, 200×, and 100×) were optimized (Figure 2E,F, respectively). The selected optimum values (CAb 2.5 µg·mL^−1^ and DAb-HRP 200×) were used for the final optimization that consisted of the WE’s nanostructuration to increase the analytical signal (Figure 2G). A bare (i.e., unmodified) SPCE was used as control, and distinct gold- and carbon-based nanomaterials were tested: gold nanoparticles (AuNP), reduced graphene oxide (rGO), carbon nanotubes (CNT), and nanodiamonds (ND). The obtained results indicated that the WE’s modification with nanomaterials was not essential to obtain a reasonable analytical signal. This avoids the use of additional materials and reagents and reduces the sensor’s cost.

Furthermore, because the temperature can influence the immunosensor’s performance [25,26], the allergen and DAb-HRP solutions were incubated at 20, 25, and 30 °C (Figure 2H), and suitable results were achieved at 30 °C.

### 3.2. Analytical Characteristics and Stability of the Immunosensor

**Figure 3:**
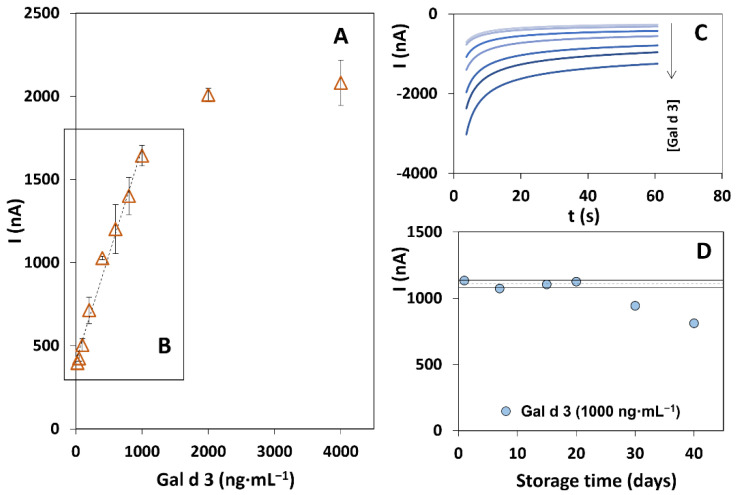
(**A**) Results of the analysis of Gal d 3 between 50 and 4000 ng·mL^−1^, (**B**) Calibration straight for Gal d 3 (55–1000 ng·mL^−1^), and (**C**) respective amperograms. (**D**) Results of the stability study (dashed line: mean values (*x*), solid lines: sum of mean and standard deviation (*n* = 3, *x* ± *φ*) of a set of measurements performed over 40 days). Other experimental parameters: chronoamperometry: 0 V, 1 min.

The analytical performance of the optimized biosensor was evaluated using Gal d 3 standard solutions with concentrations between 25 and 4000 ng·mL^−1^ (Figure 3A). A linear relationship between the current (I) and the allergen concentration was obtained between 55 and 1000 ng·mL^−1^ (I (nA) = (1.13 ± 0.03) [Gal d 3] (ng·mL^−1^) + (522 ± 18), r = 0.998, *n* = 7). Signal saturation was observed for concentrations higher than 2000 ng·mL^−1^. The calibration plot and representative amperograms are displayed in Figure 3B,C.

The limits of detection (*LOD* = 3 × S_*y*/*x*_/*m*) and quantification (*LOQ* = 10 × S_*y*/*x*_/*m*) were calculated based on the calibration plot data, where S_*y*/*x*_ is the standard deviation of the linear regression, and *m* is the slope of the calibration plot. The obtained values were LOD = 16 ng·mL^−1^ and LOQ = 55 ng·mL^−1^. The coefficient of variation of the method, calculated as *V*_x0_ (%) = S_x0_/(*x*) (where *S*_x0_ corresponds to the standard deviation of the method and *x* to the average of the concentrations of the standards), was 5.5%, demonstrating an adequate precision of the method.

Regarding the precision of the results, repeatability and reproducibility were assessed with a set of electrodes previously prepared and biomodified. To assess the repeatability, a 1000 ng·mL^−1^ Gal d 3 solution was tested using three different electrodes on the same day, obtaining a coefficient of variation (CV) of 3.8%. Reproducibility was evaluated for three consecutive days, yielding a CV of 5.7%. Therefore, as for the repeatability results, good reproducibility was achieved.

The biosensor’s stability was evaluated over several weeks, using SPCEs that were biomodified simultaneously (a 10 μL aliquot of CAb was added to each WE) and stored in a humidified container in a refrigerated environment (4–8 °C). The amperometric signal was measured after 1, 7, 15, 20, 30, and 40 days, and the obtained results (Figure 3D) demonstrate that no significant differences were observed for up to 20 days (retaining 99.4% of the initial signal), which indicates the sensor’s stability during this period. Hence, the use of previously prepared platforms reduces the volume of the reagents since a sequential preparation is carried out and also reduces the time required for the WE biomodification. After 40 days, a reduction of the amperometric signal to 71.5% of the initial signal was observed, which indicates that the sensor is still able to attain an adequate result regarding the presence/absence of the allergen, however, with a lower sensitivity.

### 3.3. Selectivity and Applicability of the Developed Immunosensor

The current EU legislation (1169/2011) does not provide a regulatory level of egg-white residues in foods and products thereof to establish critical labelling decisions and actions for adequate quality control of commercial pre-packed foodstuff from food manufacturers. Thus, besides the analytical characteristics obtained using the present biosensor (Section 3.2.), the simulation of biological samples and the evaluation of commercial samples is of utmost importance. Hence, the usefulness of the amperometric immunosensor was assessed by quantifying the egg-white allergen in spiked (fortified) allergen-free food samples or processed (commercial) samples.

**Figure 4:**
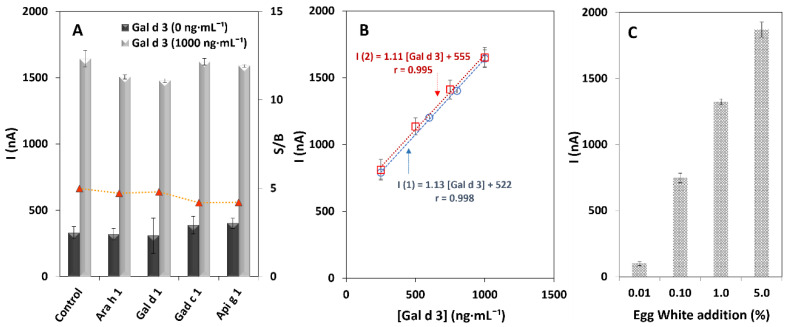
Responses provided by the amperometric immunosensor. (**A**) Selectivity study (i.e., in the absence of Gal d 3) (dark grey bars) and interference evaluation (i.e., Gal d 3 (1000 ng·mL^−1^) mixed with possible interferents) (light grey bars). Tested non-target allergens: Ara h 1, Gal d 1, Gad c 1, and Api g 1 (1000 ng·mL^−1^). (**B**) Comparison between the analysis of Gal d 3 in buffer (I1) and whole-grain extract (I2). (**C**) Addition of increasing amounts of egg white (0.010, 0.10, 1.0, and 5.0% to a food sample (whole-grain extract)).

The selectivity of the immunosensor towards Gal d 3 was tested by analysing other important food allergens that can be present in commercial food products, such as peanuts (Ara h 1), egg (ovomucoid—Gal d 1), fish (parvalbumin—Gad c 1), and celery (Api g 1). Therefore, the immunosensors’ response was studied using solutions containing the non-target proteins (1000 ng·mL^−1^) in the absence (0 ng·mL^−1^) and presence of the allergen under study (control assay, 1000 ng·mL^−1^). The selected concentrations correspond to the amount that may cause effects in hypersensitive individuals. According to the results (Figure 4A), the presence of non-target allergenic proteins generates signals similar to the blank (dark grey bars), thus demonstrating the high selectivity of the antibody pair (anti-Gal d 3). Moreover, in the presence of Gal d 3 (light grey bars), the currents were close to the ones obtained when only the target allergen was analysed. Thus, the tested non-target allergens do not interfere in the analysis. Importantly, although ovomucoid (Gal d 1) and ovotransferrin (Gal d 3) have structural similarities, no significant cross-reactivity was observed, which can be due to the chemical interaction mechanism between the analyte and the specific immuno-recognizer (allergen-specific anti-Gal d 3 antibody) [27,28].

Certified reference materials are ideally used to evaluate the accuracy of the results of an analytical method. However, because of the absence of these materials, recovery studies were carried out using a whole-grain extract selected based on its nutritional composition and the product label (“does not contain egg or any traces thereof”). The food extract was fortified with distinct concentrations of Gal d 3 within the linear calibration range (250, 500, 750, and 1000 ng·mL^−1^). Recovery percentages of 98.4, 108.6, 104.9, and 97.3% were obtained, respectively. The values are within the satisfactory range (90–110%), indicating that the biosensor provides accurate results and supports an effective detection of ovotransferrin in food samples. The CV (9.6, 5.6, 5.0, and 6.5%) once again attest to the good precision of the results. Additionally, the correlation plots presented in Figure 4B confirm that no significant matrix effects were observed since the slope obtained using the food sample (I2) and the slope of the calibration plot in buffer (I1) were similar (buffer slope/matrix slope = 1.02).

Additionally, the biosensor’s ability was assessed for the analysis of (extremely) low amounts of the egg allergen in commercial products. Thus, 1 g of whole grain was fortified with increasing percentages (0.010, 0.10, 1.0, and 5.0%) of lyophilized egg white (100% egg-white protein powder, certified product, full protein). The obtained data (Figure 4C) indicates that extremely low amounts (down to 0.010%) of the allergen can be properly detected, thus demonstrating the sensor’s effectiveness in quantifying very low amounts of egg white. Moreover, regarding the obtained results, product labels with specified egg-white amounts can be efficiently checked using the developed biosensor.

### 3.4. Analysis and Quantification of Gal d 3 in Food Samples

Egg white can be cooked and/or added as an ingredient in food products in a variety of preparations. Thus, Gal d 3 was firstly quantified in egg white obtained/prepared in the following conditions: A—liquid, B—powder, C—boiled, D—fried, E—poached, and F—whipped. Hence, the chosen samples have different physical states (e.g., liquid, solid (lyophilized)—A, B) and were processed at high temperatures (C, D) either in an acid medium (vinegar—E) or mechanically (whipped using a whisk—F). Furthermore, a whole egg (G) (yolk + white) was also analysed since the sentence “may contain traces of egg” on food labels are frequent and without specification of either the white or the yolk.

**Figure 5:**
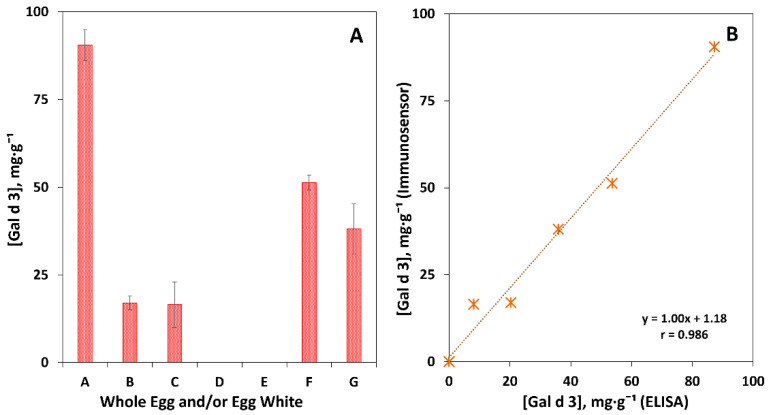
Quantification of Gal d 3 in egg. (**A**) Egg white: A—liquid, B—powder, C—boiled, D—fried, E—poached, F—whipped, and G—whole egg (yolk and white). (**B**) Correlation between the results obtained using the amperometric immunosensor and ELISA. The concentrations of the allergen (mg·g^−1^) in these samples were: (A) 90.5 ± 4.4 (CV = 4.9%), (B) 17.0 ± 1.6 (CV = 9.4%), (C) 16.5 ± 4.1 (CV = 25%), (D) <LOQ, (E) <LOQ, (F) 51.3 ± 2.2 (CV = 4.3%), and (G) 38.1 ± 3.9 (CV = 10%).

As can be seen in Figure 5A, the highest amount of Gal d 3 was quantified in sample A, which was expected, as it contains the allergen in its full/original format. In samples F and G, approximately half of the amount of sample A was found, which is due to the mechanical processing and the analysis of the whole egg, respectively. On the other hand, samples B (powdered, lyophilized to remove the liquid fraction) and C (boiled with the shell, thus without direct contact with water) showed the lowest concentrations. In samples D and E, the sensor was not able to quantify the allergen, which may be the result of the direct contact of the sample with a heat source, resulting in a differentiated protein conformation (denaturation). The processing of these samples reduces the amount of allergen to undetectable levels or even eliminates the allergen from the food sample. These results were in accordance with the ones obtained using the conventional methodology ELISA (Figure 5B). In summary, we proved that the food manufacturing/preparation processes influence the amount of allergen present in the products for consumption. In the food allergy under study (egg), allergic sensitization is caused mainly by egg-white proteins (ovomucoid, ovalbumin, ovotransferrin, lysozyme). This egg portion contains proteins that can be destroyed by heat, as is the case of ovotransferrin. However, other allergens (e.g., ovomucoid) may be resistant to high temperatures. This fact explains why some individuals only present allergic symptoms/reactions to raw egg and/or foods containing raw egg, while others do to both raw and cooked eggs (even if subjected to a high-temperature cooking process) [29]. Thus, the results in Figure 5 elucidate that individuals hypersensitive to ovotransferrin should pay attention to the cooking process, which must be carried out at high temperatures or in an acidic medium.

The sensor was also applied to analyse a wide variety of other food ingredients and/or products purchased in local supermarkets (Porto, Portugal). The selection of these products was based on the following criteria: (i) without egg, (ii) may contain traces of egg, and/or (iii) contains egg and were included in the sweets, snacks, and typical Portuguese desserts category. These traditional products are generally cooked using both egg white and egg yolk and are highly consumed. Besides this, other Mediterranean products that are part of daily meals (e.g., vegetable broths, pasta, etc.) were also tested.

The food products were grouped into distinct categories. Egg-Free: 1—whole-grain cereal, 2—wafer, 3—wheat Flour, 4—celery, 5—sesame seed, 6—soybean, 7—peanut, 8—hazelnut, 9—oat, 10—royal pudding, and 11—powdered flan pudding; may contain traces of egg: 12—peanut and pineapple biscuit, 13—water and salt cracker, 14—Portuguese cookie (maria), 15—vegetable broth, and 16—powdered “Pão de Ló”; and contains egg and/or contains egg white: 17—biscuits, 18—muffins (egg 13%, egg white 8%), 19—sponge cake (egg 5%, egg white 8%), 20—pasta (60% of wheat semolina + egg white), 21—cake roll (egg 7%, egg white 3%), 22—flan pudding (egg 17.8%), 23—egg pudding (egg 5%), 24—“Pão de Ló” (powdered egg), and 25—“Doce de Ovos” (egg 13%). The results of the analyses, using the immunosensor and an ELISA assay, are presented in Table 1. The sample preparation procedure is reported in Materials and Methods (the sample extracts were diluted 250× using B1-BSA).

In the “egg-free” section (samples 1–11), besides ingredients mentioned on food product labels, several major food allergens included in EU Regulation 1169/2011 were analysed to verify the selectivity of the immunosensor. Additionally, foodstuffs in which the original recipe contains egg (wafer, royal pudding, powder flan pudding) but whose label does not mention the presence of egg were also included. Gal d 3 was only found in sample 2 (38.5 ± 3.2 µg·g^−1^). The label of this product does not comply with legislation because there is no mention of the (possible) presence of egg. Thus, the results show the feasibility of the developed method to track Gal d 3 even at trace amounts.

In turn, in the “may contain traces of egg” category (samples 12–16), Gal d 3 was found in all the samples except for sample 15. This validates the product labels. The label of sample 15 is probably precautionary to comply with the legislation, which may be due to the fabrication of the product on equipment where samples containing egg are also processed.

For the “contains egg” and/or “contains egg white”” set of samples (samples 17–25), distinct results were obtained. This may be in accordance with the cooking method (discussed previously). In samples 17 (containing egg), 18 (egg 13%, egg white 8%), and 19 (egg white 8%, egg 5%), despite the reference to egg and/or egg white, Gal d 3 was not quantifiable with the immunosensor. This can be the result of the production processes in which an oven at temperatures higher than those of cooking in water was used. In samples 20 (60% wheat semolina, egg white), 21 (egg 7%, egg white 3%), and 22 (egg 17.8%), Gal d 3 was quantifiable; however, the obtained concentration was lower than expected. Sample 20 was cooked in a water bath, and sample 21 was a paste previously baked in an oven with the subsequent addition of a cream containing egg white. The label of sample 22 did not state whether the egg content represented the whole egg, the yolk, and/or the egg white; thus, it may not contain egg white. Therefore, despite food processing resulting in a decrease of the allergen content due to possible denaturation of the protein/allergen, it is possible to quantify low concentrations of Gal d 3. Samples 23 (egg 5%), 24 (egg powder), and 25 (egg 13%) revealed high amounts of Gal d 3. These samples are provided in packages with lyophilized content containing egg powder, but the real content (white and/or yolk) is not mentioned. The results of the analyses of the samples using the immunosensor and a conventional method (ELISA) are indicated in Table 1.

Interestingly, not in all products was the allergen found using both the immunosensor and the ELISA method. The latter method was not able to quantify the allergen in several samples (e.g., wafer, peanut and pineapple biscuits, pasta). Moreover, the obtained data are in accordance with the previously reported results for the analysis of egg-white allergens in food products [5,30], thus refuting the supposition that undeclared residues of eggs can be found in food products (without precautionary label), as their presence can result from possible uncontrolled/unknown contamination (e.g., transport, storage, raw materials). A summary of the analytical methodologies developed for Gal d 3 analysis in food samples and their comparison with total egg-white protein is shown in Table 2 [5,30,31]. The present sensor can be used for individual analysis at the sampling site and in a shorter time (30 min) compared to those described in the literature (>2 h), which also require expensive equipment and specialized technicians.

To the best of our knowledge, the present work reports the first electrochemical immunosensor for the analysis of Gal d 3 in pre-packed foodstuff. According to the analytical characteristics reported for the few electrochemical biosensors concerning equally important egg-white allergens (ovomucoid (Gal d 1) [12] and ovalbumin (Gal d 2) [13,14,15,16,17]), screen-printed electrodes were preferred as transducer since reduced volumes and portable systems are used except for [16], which uses conventional electrodes, and thus, a greater volume of sample/solution is required. Moreover, contrary to our simple and feasible work, the previously published ones use nano/micro materials with a laborious, time-consuming, and expensive transducer preparation/nanostructuration involving additional reagents. Among the electrochemical techniques, voltammetry and amperometry stand out, and because no amperometric immunosensors for the analysis of Gal d 3 are reported, the obtained figures of merit are not comparable since each allergen has different characteristics.

Another advantage of this work is the fact that the developed biosensor was applied to the analysis of many commercial food samples when compared to the previously published works whose application is scarce: chicken eggs and bread [12], biscuits [13], cake [14], and wine [17].

## 4. Conclusions

The present amperometric immunosensor was developed for the difficult task of tracking Gal d 3—a major egg-white allergen—in commercial foodstuff. In total, 7 egg preparations and 25 food samples were analysed. With an assay time of 30 min, a hands-on time of less than 10 min, and an amperometric measurement of 1 min, a sensitive biosensor was developed using a sustainable, easy-to-use, and portable platform. The sensor’s selectivity towards the target protein, its stability (over 20 days), and applicability to pre-packaged products prove that it can be a useful analytical tool for food producers, commercial establishments, and regulatory agencies.

## Figures and Tables

**Table 1:** Results of the analysis of ovotransferrin (µg·g^−1^) in 25 ingredients and/or food products using the developed amperometric immunosensor and an ELISA assay.

Food/Ingredient		Gal d 3 (µg·g^−1^)			
		Immunosensor	CV (%)	ELISA	CV (%)
Egg-Free	1-Whole grain cereal	<LOQ	–	<LOQ	–
	2-Wafer	38.5 ± 3.2	8.4	<LOQ	–
	3-Wheat flour	<LOQ	–	<LOQ	–
	4-Celery	<LOQ	–	<LOQ	–
	5-Sesame seed	<LOQ	–	<LOQ	–
	6-Soybean	<LOQ	–	<LOQ	–
	7-Peanut	<LOQ	–	<LOQ	–
	8-Hazelnut	<LOQ	–	<LOQ	–
	9-Oat	<LOQ	–	<LOQ	–
	10-Royal pudding	<LOQ	–	<LOQ	–
	11-Powdered flan pudding	<LOQ	–	<LOQ	–
May Contain Egg	12-Peanut and pineapple biscuit	108.4 ± 10.1	9.3	<LOQ	–
	13-Water and salt crackers	80.1 ± 5.8	7.2	<LOQ	–
	14-Portuguese cookie (maria)	112.4 ± 3.0	2.7	<LOQ	–
	15-Vegetable broth	<LOQ	–	<LOQ	–
	16-Powdered “Pão de Ló”	115.0 ± 4.2	3.7	<LOQ	–
Contain Egg and/or Egg White	17-Biscuits (egg)	<LOQ	–	12.9 ± 1.7	13.1
	18-Muffins (egg 13%, egg white 8%)	<LOQ	–	<LOQ	–
	19-Sponge cake (egg 5%, egg white 8%)	<LOQ	–	<LOQ	–
	20-Pasta (60% of wheat semolina + egg white)	48.7 ± 3.7	7.6	<LOQ	–
	21-Cake roll (egg 7%, egg white 3%)	362.8 ± 21.5	8.7	307.1 ± 4.2	1.4
	22-Flan pudding (egg 17.8%)	112.8 ± 4.9	4.3	112.1 ± 2.3	2.1
	23-Egg pudding (egg 5%)	2376 ± 15.8	0.7	2242 ± 4.5	0.2
	24-“Pão de Ló” (powdered egg)	2646 ± 23.8	0.9	2436 ± 18.2	0.7
	25-“Doce de Ovos” (egg 13%)	1995 ± 42.7	2.1	1755 ± 10.1	0.6

**Table 2:** Summary of analytical methods for egg-white allergen detection.

Analyte	Methodology		Assay Time	Analytical Features		Application	Ref
	Method	Technique		Linear Range	LOD		
Gal d 3	Immunosensor	Electrochemistry	30 min	55–1000 ng·mL^−1^	16 ng·mL^−1^	Yes	This work
	MIP	Microfluidics	<2 h	n.r.	n.r.	No	[31]
	ELISA	Spectrometry	>3 h	n.r.	n.r.	Yes	[30]
Egg-white protein	ELISA	Spectrometry	>2 h	0–24 µg·g^−1^	0.25 µg·g^−1^	Yes	[5]

n.r., not reported; Gal d 3, ovotransferrin; ELISA, Enzyme-Linked Immunosorbent Assay; MIP, molecularly imprinted polymer.

## Data Availability

Not applicable.
